# Voluminous Pancreatic Mucinous Cystadenoma in a Non-Pregnant Woman with Rheumatoid Arthritis

**Published:** 2011-08-01

**Authors:** S R Modarres, S Siadati, Z Momeni

**Affiliations:** 1 Department of Surgery, Babol University of Medical Sciences, Babol, Iran; 2 Department of Pathology, Babol University of Medical Sciences, Babol, Iran; 3 Clinical Research Development Center, Shahid Beheshti Hospital, Babol, Iran

**Keywords:** Mucinous cystadenoma, Pancreas, Rheumatoid arthritis

**Fig. 1:**
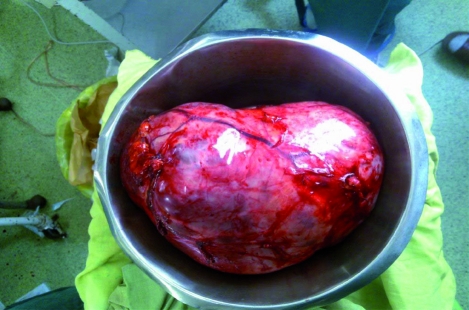
A huge cystic mass was found originating from body to tail of the pancreas.

Dear Editor,

Indeed, cystic tumors of the pancreas are rare and account for 10–15% of pancreatic neoplasms.[1] There are four main categories of pancreatic cystic tumors including i) Mucinous cystic tumors (MCTs); ii) Serous cystic tumors (SCTs); iii) Intraductal papillary cystic tumors (IPCTs); and iv) Papillary cystic tumors. [2][3]Mucinous cystic neoplasm of the pancreas is an uncommon tumor characterized by an inner mucin-producing columnar epithelium layer and an outer dense cellular ovarian-like stromal layer. These tumors are typically localized in the body and tail of the pancreas and do not communicate with the pancreatic ductal system. [1][4] According to the studied reported so far, MCTs are found more commonly in females, especially in middle-aged more often than the elderly, and therefore are sex hormone- sensitive. [3][4] Most of the large MCTs reported to date were in pregnant women and supposed to be due to over expression of sex hormones during this period. [5][6][7][8][9]This is the first report of a huge MCT in a non-pregnant woman with rheumatoid arthritis having been under corticosteroids treatment. A 50-year-old woman was referred to Shahid Beheshti Hospital, Babol, Iran because of abdominal pain, distention and left abdominal mass. The patient was non-alcoholic with no history of gallbladder or pancreatitis. She had only a history of rheumatoid arthritis diagnosed at 42 years of age and had been under prednisolone (a corticosteroid) treatment. Physical examination suggested the presence of a mass in the left hypocondrium. Abdominal CT scan proved this finding. The patient underwent tumor resection with distal pancreatectomy due to the size and difficult handling of the tumor. A voluminous mass was found originating from the pancreatic body-tail (Figure 1). No evidence of invasive or metastatic tumor spread was observed within the abdomen. Pathological examination showed a 23×11×14 cm multilocular tumor, weighted 3065 g and pinkish gray. The cyst had a smooth internal surface filled with dark brown fluid. Microscopically, the cyst wall lined by mucin-producing columnar epithelium associated with an outer dense cellular ovarian- like stroma. The histological diagnosis was a mucinuos cystic neoplasm (musinous cystadenoma). The postoperative course was uneventful and the patient discharged 6 days after surgery in good general condition. After 6 months follow up, the patient was in good health and presented no signs of recurrence. However, the unusual size of the tumor was in contrast to the findings from other reports, since the patient was not pregnant with the cyst showing no sign of malignancy. Up to now, most of the large reported MCTs were in pregnant women and supposed to be due to over expression of sex hormones during this period. [5][6][7][8][9]This is the first case of a huge MCT in a nonpregnant woman with rheumatoid arthritis having been under corticosteroids treatment. There is also another case reporting a large MCT in the appendix of a rheumatoid arthritis patient treated with steroids.[10] To date, there is less done to identify the presence of various steroid receptors in MTNs. These studies indicate estrogen and progesterone receptors in the mass.[11][12][13] Since the presence of other steroid receptors, especially glucocorticoid receptors has not been proven in MCTs, it is likely that prednisolone cause irregular mass overgrowth by interacting with its possible glucocorticoid receptors. In this regard, over expression of glucocorticoid receptors in human pancreatic cancer has been shown in one study.[14] Therefore we suggest that steroids side effects, especially in patients with potentially malignant tumors, be investigated further.
